# Introduction of the levonorgestrel intrauterine system in Kenya through mobile outreach: review of service statistics and provider perspectives

**DOI:** 10.9745/GHSP-D-13-00134

**Published:** 2014-01-09

**Authors:** David Hubacher, Vitalis Akora, Rose Masaba, Mario Chen, Valentine Veena

**Affiliations:** a FHI 360, Research Triangle Park, NC, USA; b Marie Stopes/Kenya, Nairobi, Kenya

## Abstract

**Background:**

The levonorgestrel intrauterine system (LNG IUS) was developed over 30 years ago, but the product is currently too expensive for widespread use in many developing countries. In Kenya, one organization has received donated commodities for 5 years, providing an opportunity to assess impact and potential future role of the product.

**Methods:**

We reviewed service statistics on insertions of the LNG IUS, copper intrauterine device (IUD), and subdermal implant from 15 mobile outreach teams during the 2011 calendar year. To determine the impact of the LNG IUS introduction, we analyzed changes in uptake and distribution of the copper IUD and subdermal implant by comparing periods of time when the LNG IUS was available with periods when it was not available. In addition, we interviewed 27 clinicians to assess their views of the product and of its future role.

**Results:**

When the LNG IUS was not available, intrauterine contraception accounted for 39% of long-acting method provision. The addition of the LNG IUS created a slight rise in intrauterine contraception uptake (to 44%) at the expense of the subdermal implant, but the change was only marginally significant (*P* = .08) and was largely attributable to the copper IUD. All interviewed providers felt that the LNG IUS would increase uptake of long-acting methods, and 70% felt that the noncontraceptive benefits of the product are important to clients.

**Conclusions:**

The LNG IUS was well-received among providers and family planning clients in this population in Kenya. Although important changes in service statistics were not apparent from this analysis (perhaps due to the small quantity of LNG IUS that was available), provider enthusiasm for the product was high. This finding, above all, suggests that a larger-scale introduction effort would have strong support from providers and thus increase the chances of success. Adding another proven and highly acceptable long-acting contraceptive technology to the method mix could have important reproductive health impact.

## Abstract (precis)

Limited introduction of the LNG IUS through mobile outreach in Kenya, without any special promotion, resulted in good uptake. And providers viewed it positively, particularly because of its noncontraceptive benefits. Increased provision of the LNG IUS can improve options for women needing highly effective reversible contraception.

## INTRODUCTION

In many countries, the levonorgestrel intrauterine system (LNG IUS) has become an important reproductive health commodity. The most recent example is from the United States, where it was approved 13 years ago. The LNG IUS is now more popular in the United States than any new contraceptive product introduced since 1992, including the DMPA (depot medroxyprogesterone acetate) injectable (1992), the vaginal ring (2001), the contraceptive patch (2002), and the etonogestrel implant (2006).^1^^–^^3^ In resource-poor countries, the commercial LNG IUS product may be available in the private sector, but only the highest socioeconomic classes can realistically afford it. In Kenya, for example, the commercial product costs approximately US$200.

Cost is not the only consideration when contemplating the potential role and impact of the LNG IUS. Although the LNG IUS is a form of intrauterine contraception, as is the copper intrauterine device (IUD), it should not be viewed as simply another IUD. The LNG IUS and the copper IUD have striking differences in side effects and noncontraceptive benefits. However, in terms of effectiveness and other factors, the products have important similarities as well (Table 1).^4^^,^^5^

**TABLE 1.** Comparing the LNG IUS and the Copper IUD

**Points of Comparison**	**LNG IUS**	**Copper IUD**
Main difference	Hormonal (levonorgestrel)	Nonhormonal
Main effects on menstruation	Generally *decreases*menstrual blood loss	Generally *increases*menstrual blood loss
Duration of use	5 years	10–12 years
Noncontraceptive benefits (stemming from reduced uterine bleeding)	Treats menorrhagia, increases hemoglobin, and likely alleviates anemia and problems with uterine fibroids	…
Effectiveness	Both in the highest tier of contraceptive effectiveness	
Continuation rates	Both have similar, high continuation rates	

Abbreviations: IUD, intrauterine device; LNG IUS, levonorgestrel intrauterine system.

A noncommercial LNG IUS product is currently being introduced on a very limited basis into some programs in resource-poor countries through donations from the International Contraceptive Access (ICA) Foundation (a partnership between Bayer Healthcare Pharmaceuticals and the Population Council). Since inception in 2003, the ICA Foundation has donated nearly 50,000 LNG IUS devices to 19 countries.^6^ In recipient programs, the LNG IUS is provided free-of-charge alongside established contraceptives so women can have expanded choice. Programs are embracing the donations so their clients can benefit from state-of-the-art contraception.

When new contraceptives become available, they generally improve reproductive health in the affected population. In a multicountry analysis, Jain found that overall contraceptive use rises with increased method choice,^7^ and a review of international data over 27 years showed that as each additional contraceptive method became available to most of the population, overall modern contraceptive use rose.^8^ A systematic review found that increased choice raises contraceptive uptake, improves health outcomes, and improves adherence.^9^ In contrast, Sutherland et al. analyzed data from 13 countries and found that the rise in injectable use was partially offset by declines in use of other methods,^10^ whereas Ross found macro-level evidence that expansion of injectables attracted new users to family planning.^11^ Recent introduction of a natural family planning method resulted in sizeable uptake in 3 country programs.^12^

The more contraceptive methods available, the greater the contraceptive prevalence generally.

At the family planning program level, it is important to know how a new product might alter the pattern of method uptake and services. For example, a new method might attract new clients and overburden the service delivery system, particularly if the new method requires more time and effort to provide. If a new method becomes popular, a program will need to purchase enough product to satisfy demand, and potentially decrease orders of other products if a contraceptive substitution effect occurs.

During any product introduction process, providers' opinions are critical. Given their important role in contraceptive counseling,^13^ providers can help shape the impact of a new product. Previous research has shown that family planning counselors are more likely to recommend methods that they use themselves,^14^ and client uptake of methods is also linked to providers' personal method use.^15^ Similar patterns have been seen in the use of hormone replacement therapy.^16^ In many settings, decades can pass before initial introduction leads to widespread national availability.

Since 2008, the Marie Stopes affiliate in Kenya (MSK) has provided free LNG IUS services to approximately 5,000 women. Thus, during this time, some clients had one additional long-acting reversible contraceptive (LARC) choice, which also includes subdermal implants and all types of intrauterine devices. We undertook this project at MSK to better understand the impact of the LNG IUS introduction efforts.

## METHODS

We used anonymous MSK service statistics and interviews with MSK providers to evaluate the LNG IUS introduction activity. This research was approved by the Protection of Human Subjects Committee (of FHI 360) and the Kenya Medical Research Institute's Ethical Review Committee. The MSK providers voluntarily agreed to be interviewed through an informed consent process that was approved by these committees.

The LNG IUS product was used in MSK's outreach program, which consists of 15 teams in different geographic regions of Kenya. Each team has 2 clinicians (1 medical doctor and 1 nurse) and 2 care assistants; the teams visit catchment public-sector health facilities on a rotating basis to provide family planning services.

We reviewed MSK's existing (internal) reporting systems to tabulate the monthly number of contraceptive method insertion procedures for each of the 15 outreach teams during the 2011 calendar year. We developed an electronic database and entered into a spreadsheet the number of insertions for the LNG IUS, copper IUD, and subdermal implant. Of the 15 teams, 4 did not receive any LNG IUS in the study period and were excluded from the analysis. Of the possible 132 total available months across the 11 teams, 2 months with no LARC insertions (all 3 methods combined) were excluded from the calculations. Thus, a total of 130 months of data were included in the analysis.

For each team, we computed the mean number of monthly product insertions. In some months and in some teams, the LNG IUS was not available. Thus, we examined how the average proportions of total IUD (copper IUD plus LNG IUS) versus subdermal implant insertions varied per month, by whether or not the LNG IUS was available. We used a *t* test to determine whether availability of the LNG IUS increased total IUD insertions, relative to the subdermal implant.

For feedback on the LNG IUS product, we interviewed 27 MSK providers. We asked a variety of questions to characterize their views and their clients' views on the new product.

## RESULTS

During the 2011 calendar year, the outreach program provided over 67,000 women with LARCs: 39% chose the copper IUD, 60% the subdermal implant, and 1% the LNG IUS (Table 2). During this time period, 11 outreach teams provided a total of 1,030 LNG IUS insertions. On a monthly basis, the mean number of insertions per team varied considerably for each product: 201 for the copper IUD, 309 for the subdermal implant, and 8 for the LNG IUS.

**TABLE 2.** LARC Insertions Performed by 11 Outreach Teams of Marie Stopes/Kenya, 2011

**Device**	**Total No.**	**Mean^a^ (range)**
Copper IUD	26,070	201 (20, 509)
Subdermal implants	40,146	309 (25, 772)
LNG IUS	1,030	8 (0, 116)

Abbreviations: IUD, intrauterine device; LARC, long-acting reversible contraceptive; LNG IUS, levonorgestrel intrauterine system.

a Per outreach team, per month.

During months when the LNG IUS was not available, IUD services accounted for an average of 38.9% of total LARC services (Table 3). The average proportion of women selecting an IUD (copper IUD or LNG IUS) rose slightly (to 44.3%) when the LNG IUS was available, but the change was only marginally significant (*P*  = .08). The modest increase was largely attributable to the high volume of copper IUDs relative to the volume of the LNG IUS. The relative importance of the IUD versus the implant varied considerably across the different teams, regardless of whether the LNG IUS was available.

The average proportion of women selecting either a copper IUD or LNG IUS rose slightly when the LNG IUS was available.

**TABLE 3.** Mean Number of Monthly LARC Insertions and Proportion of IUD Insertions per Marie Stopes/Kenya Outreach Team, by LNG IUS Availability,^a^ 2011

**Team**	**LNG IUS Not Available**				**LNG IUS Available**					
	**No. of Months**	**Implants**	**Copper IUD**	**% IUD^b^**	**No. of Months**	**Implants**	**Copper IUD**	**LNG IUS**	**Total IUD^c^**	**% Total IUD^b^**
1	4	566	227	29.5	8	424	242	5	247	37.3
2	10	329	165	38.3	1	416	147	3	150	26.5
4	5	273	164	43.5	6	352	138	9	147	29.5
7	8	278	159	37.4	4	368	174	3	177	32.8
8	1	285	193	40.4	11	227	128	14	142	43.0
9	10	447	188	31.4	2	306	205	10	215	42.7
11	6	391	215	33.5	6	408	291	9	300	41.9
12	2	147	268	66.4	10	229	281	38	319	60.7
13	6	254	310	59.1	6	328	314	14	328	52.1
14	2	185	486	72.4	10	181	205	19	224	54.9
15	9	210	72	26.9	3	273	45	10	55	19.2
Total	63	322	191	38.9	67	296	210	15	225	44.3

Abbreviations: IUD, intrauterine device; LARC, long-acting reversible contraceptive; LNG IUS, levonorgestrel intrauterine system.

a *P* value = .08 for testing whether the relative monthly distribution of IUD vs. implants is different depending on availability of the LNG IUS.

b Mean of the proportion of women receiving IUDs per month.

c Total IUD is the sum of copper IUD and LNG IUS insertions.

In the survey of MSK providers, varying experiences with the LNG IUS were noted (Table 4). For example, nearly half of the MSK providers inserted 51 or more LNG IUS while 11% had not inserted even 1 device. About half of providers felt equally comfortable describing and providing all 3 long-acting methods (the implant, the copper IUD, and the LNG IUS). For the half who were not equally comfortable with describing/providing all 3 long-acting methods, they were most comfortable with the subdermal implant and least comfortable with the LNG IUS. All respondents felt that the LNG IUS would attract new clients to long-acting methods, and 70% believed that the noncontraceptive benefits of the product were very important to their clients.

All interviewed providers felt that the LNG IUS would attract new clients to long-acting methods.

**TABLE 4.** Experiences With the LNG IUS Among Marie Stopes/Kenya Providers (N = 27)

**Provider Experiences**	**% Distribution**
Number of LNG IUS insertions performed since completing training	
0	11
1–10	26
11–50	15
51+	48
Method *most* comfortable describing and providing	
Copper IUD	0
Subdermal implant	48
LNG IUS	0
All the same	52
Method *least* comfortable describing and providing	
Copper IUD	4
Subdermal implant	0
LNG IUS	44
All the same	52
Do you think clients easily understand the difference between the copper IUD and the LNG IUS?	
No	30
Yes	70
Is the 10+ years duration of use for the copper IUD a significant reason women will choose it instead of the LNG IUS that only lasts for 5 years?	
No	52
Yes	44
Do not know	4
Did you ever have a stockout of the LNG IUS?	
No	4
Yes	96
If the LNG IUS is not available, what method do women choose instead?	
Subdermal implant	70
Copper IUD	30
Other	0
Will the LNG IUS attract new clients to long-acting contraception?	
No	0
Yes	100
How important are noncontraceptive benefits of the LNG IUS to your clients?	
Very important	70
Somewhat important	30

Abbreviations: IUD, intrauterine device; LNG IUS, levonorgestrel intrauterine system.

In distinguishing the LNG IUS from the copper IUD, providers cited these main features: hormonal product (85%), reduces bleeding (52%), and 5-year duration of use (44%) (Table 5). Nearly 60% of providers cited reduction in menstrual bleeding as a key “attractive” attribute for clients. Forty-four percent of providers reported that the hormonal content of the product is a feature that clients find unattractive. Reduction of menstrual blood loss was the primary noncontraceptive benefit reported by 52% of providers.

**TABLE 5.** Main Attributes of LNG IUS Cited by Marie Stopes/Kenya Providers (N = 27)

**Attribute**	**% Distribution**
Key information provided to clients to distinguish the LNG IUS from the copper IUD^a^	
Hormonal product	85
Reduces bleeding	52
Duration of use	44
Works locally in uterus	15
Prevents cancer	15
Aspects of the LNG IUS that are *attractive* to clients^a^	
Reduction of excessive menstrual bleeding	59
5-year product	15
Hormonal effect	15
Plastic/nonmetallic	15
Duration of use	15
Aspects of the LNG IUS are *unattractive* to clients^a^	
Contains hormones	44
Insertion procedure/modesty issues	22
Strings cause discomfort during sex	19
What are the most important noncontraceptive benefits of the LNG IUS?^a^	
Reduces menstrual blood loss	52
Prevents cancer/uterine disease	22
Prevents anemia	22
Treats heavy menstrual blood loss	22
Alleviates pain during menses	15

Abbreviations: IUD, intrauterine device; LNG IUS, levonorgestrel intrauterine system.

a Multiple responses allowed; only responses garnering at least 15% (n = 4) are shown.

## DISCUSSION

Introduction of the LNG IUS through the Marie Stopes/Kenya outreach program had mixed impact. On the one hand, availability of the LNG IUS did not appear to alter provision of standard long-acting reversible methods (the subdermal implant and the copper IUD); the pattern of service statistics did not change for the program as a whole and for most of the outreach teams that provided the LNG IUS. However, MSK providers unanimously believed the LNG IUS would attract new clients to long-acting methods, at least partly due to the important and unique noncontraceptive benefits that the technology offers. The high volume of copper IUD services (typical for this program) demonstrated high acceptability of this product among both providers and clients. Thus, this is another example of how dedicated LARC providers are successful at making important technologies available.

Dedicated LARC providers can help improve access to contraceptive methods.

A previous introduction assessment of the LNG IUS in Ghana had similar results to ours.^17^ For example, availability of the product did not significantly alter provision of other methods. (However, the small quantity of product may have made this difficult to assess adequately.) Another similarity was found in terms of positive provider feedback about the LNG IUS; all Ghanaian providers found the product easy to insert, and all stated that their clients were satisfied with it. A more general assessment of the global LNG IUS donation activities highlighted the importance of working with in-country “product champions” and committed service-delivery counterparts with IUD insertion experience.^18^

Providers in our study candidly reported being most comfortable describing and providing the subdermal implant; this finding exposes some of the challenges for wider provision of intrauterine contraception. Reasons for being more comfortable with subdermal implants could simply be a function of higher client demand and thus more frequent counseling about and insertion of implants. However, if providers are reluctant to offer intrauterine contraception, due to perceived lack of expertise or for other reasons, a feedback loop of diminished contraceptive choices could develop. Although there is certainly no evidence that this is occurring at MSK, it is critical that providers maintain skills and confidence with all LARCs.

A larger contemporary concern across sub-Saharan Africa is widespread absence of LARC services in public-sector settings, where providers and health systems as a whole typically rely on provision of short-acting methods. Providers are subject to many personal and external influences that can ultimately limit contraceptive choice for their clients.^19^

### Limitations and Strengths of the Study

Our study in Kenya had important limitations. First, this introduction of the LNG IUS was not conducted in a promotional or scientifically rigorous way to measure the impact. For example, clients were probably not aware of the product until they spoke to the provider; thus, it is not possible to conclude anything about true demand for the LNG IUS. However, it is feasible that unmeasured word-of-mouth could have prompted some women to visit the clinic on MSK outreach days. Second, we did not conduct an experiment on the impact of the product introduction; the work was retrospective and observational of a program and not of a controlled intervention. Lastly, the quantity of LNG IUS (1,030 units) was very small relative to the other products (over 66,000 units); if unlimited supplies of the LNG IUS were available, it is possible that a different picture would have emerged. For purposes of the analysis, we assumed that zero LNG IUS insertions in a given month meant that the product was not available at that time.

The major strength of this LNG IUS introduction project is that it was done in a natural program setting, where clinicians simply offered the new product without a research aim or protocol. Also, MSK had several years of experience with the LNG IUS before data collection and interviews took place. Thus, perhaps the provider feedback is a more experienced and reflective view of the LNG IUS technology.

## CONCLUSION

The LNG IUS was developed in the 1970s and is long overdue for introduction into resource-poor settings. High product cost is the current barrier to more widespread use. New LNG IUS products made by Indian companies are currently available in India,^20^^,^^21^ and a U.S.-based company is currently seeking approval from the U.S. Food and Drug Administration for its version of the LNG IUS technology.^22^ These products hopefully will become available to resource-poor countries at a reasonable cost to international donor agencies. In summary, the results from this study suggest that the LNG IUS will have provider support and enthusiasm, which in turn can improve options for women needing highly effective reversible contraception.
